# Minimizing marine ingredients in diets of farmed Atlantic salmon (*Salmo salar*): Effects on growth performance and muscle lipid and fatty acid composition

**DOI:** 10.1371/journal.pone.0198538

**Published:** 2018-09-21

**Authors:** Maryam Beheshti Foroutani, Christopher C. Parrish, Jeanette Wells, Richard G. Taylor, Matthew L. Rise, Fereidoon Shahidi

**Affiliations:** 1 Department of Ocean Sciences, Memorial University of Newfoundland, St. John’s, NL, Canada; 2 Cargill Innovation Center, Dirdal, Norway; 3 Department of Biochemistry, Memorial University of Newfoundland, St. John’s, NL, Canada; Universidade de Vigo, SPAIN

## Abstract

Due to limited fish meal and fish oil resources and their high costs for the aquaculture industry, it is necessary to find alternative sustainable sources of protein and lipids. Therefore, seven different diets were formulated with different levels of animal by-products, vegetable proteins, fish oil and rapeseed oil, to feed farmed Atlantic salmon, and their effects on growth performance, muscle lipid class, and fatty acid composition were examined. Protein sources included anchovy, poultry, feather, blood, corn, soy and wheat. Growth performance indicated that the diet with the lowest fish meal and fish oil content resulted in the lowest weight gain and final weight, followed by the diet containing the highest level of animal by-products. The lipid class analysis showed no statistical difference in the muscle total lipid content using different diets. However, significant statistical differences were observed among the main lipid classes; triacylglycerols, phospholipids, and sterols. The diet containing 1.4% omega-3 long-chain fatty acids resulted in the highest content of triacylglycerols and phospholipids. Diets containing medium and low levels of fish oil and fish meal, respectively, led to as high a level of ω3 fatty acids in muscle as when fish were fed diets with high levels of fish meal and fish oil. The results of this study suggest that feeding a diet containing low levels of fish meal and moderate levels of fish oil does not significantly affect ω3 fatty acid composition in muscle. Fish meal could be reduced to 5% without affecting growth as long as there was a minimum of 5% fish oil, and animal by-products did not exceed 26% of the diet.

## Introduction

From 1995 to 2004, global consumption of fish meal and fish oil approximately doubled [1]. However, production of fish meal has not changed significantly in the past 30 years, and fish oil has been produced at much lower levels [2]. Fish meal contains valuable protein with high digestibility as well as essential vitamins and minerals [3]. Fish oil is a valuable primary source of lipid providing beneficial omega (ω)-3 polyunsaturated fatty acids (PUFA). Aquaculture’s high reliance on fish oil has prompted study into alternative lipid sources to reduce pressure on consumption of fish oil [4]. However, finding sustainable ingredients that could provide the required nutrition for fish has been difficult as these alternative sources are not as nutritious as fish oil [5].

Marine fish and salmonids require arachidonic acid (ARA, 20:4ω6) and high levels of essential omega-3 fatty acids, eicosapentaenoic acid (EPA, 20:5ω3) and docosahexaenoic acid (DHA, 22:6ω3), as they cannot synthesize them easily. However, fish have some ability to supply these essential fatty acids by synthesizing them from shorter chain PUFAs (e.g. 18:3ω3 and 18:2ω6). Therefore, it is important that high levels of PUFAs are available in the diet [6].

EPA and DHA are supplied to humans by fish and they play an important role in different functions, including the nervous system, photoreception, reproduction, as well as reducing the risk of cardiovascular and inflammatory diseases [7]. Therefore, it is important to ensure that these essential fatty acids are provided in fish diets as they contribute to the health of fish and the human consumer. Growth, flesh quality, and health of fish are improved as dietary levels of EPA and DHA increase [6].

Proteins constitute 70% of fish tissue dry weight. Hence, protein and essential amino acid content in fish diets play a key role in growth performance and production of high quality fish. However, minimizing use of protein is important as it is one of the most expensive components of the feeds.

Higher costs and limited availability of fish meal and fish oil have led to several evaluations of the effect of their replacement with alternative sustainable sources, such as those derived from terrestrial plants and animals in diets of different fish species. While partial replacement generally was successful in terms of growth performance, full replacement mostly resulted in lower content of ω3 and essential fatty acids, as well as poor growth performance [8], [9], [10], [11].

Several studies have evaluated the effect of replacing fish meal and fish oil with terrestrial plant sources in feeds for farmed animals [12] as they are sustainable sources of energy for fish growth that are readily available at lower prices [13], [14]. However, one problem that limits full replacement of fish oil with plant oils is the low level of ω3 fatty acids and high levels of ω6 and ω9 fatty acids. Plant oils can provide the same relative ratios of saturated fatty acids, monounsaturated fatty acids (MUFA) and PUFA as found in fish oil, but not the level of highly unsaturated fatty acids in fish oil [15], [16]. Nonetheless plant oils can replace a major amount of fish oil in the diets [8], [9], [10], [11] with no significant influence on fish growth as long as sufficient levels of essential fatty acids are provided [12]. To minimize any reduction in growth, and nutritional quality in terms of health benefits of farmed fish to human consumers, potential substitutes for fish oil should avoid excessive deposition of 18:2ω6, retain high levels of ω3 PUFA and provide sufficient energy in the form of saturates and MUFA [17]. It is claimed that as long as essential fatty acid requirements are met, fish oil can be substantially replaced by MUFA-rich alternative sources. Rapeseed oil is one of the best candidates to replace fish oil because its MUFA are 55–72% of the fatty acids [11]. MUFA cannot be converted to essential fatty acids like LA, ALA, and ω3 long-chain PUFA, so the main reason for using plant oils rich in MUFA is their ability to provide energy [11].

Replacing fish oil with rapeseed oil in diets of Atlantic salmon (*Salmo salar*) on tissue lipid composition showed no effect on growth performance or feed efficiency [13]. However, high lipid levels occurred in the liver in fish fed 100% rapeseed oil, and replacement at 50% or higher significantly reduced levels of 20:5ω3, 22:6ω3, and the ratio of ω3 to ω6 in the muscle making it less beneficial for humans.

Other alternative sources to replace fish meal are terrestrial plant proteins [9] and rendered terrestrial animal products [18], [19]. Animal by-products are widely used in diets given to carnivorous species because they prefer replacements of animal origin due to palatability. Their use has several advantages, including being free from antinutritional factors such as phytic acid, phosphorus, or indigestible complex carbohydrates. Furthermore, they contain low amounts of carbohydrates, and are high in crude protein and crude lipids, as well as vitamins and trace minerals [20]. In addition to the good nutritional value of poultry by-product meal and hydrolyzed feather meal, they have a very competitive cost advantage over fish meal [21].

While there have been several studies on replacing marine ingredients with alternative sustainable sources, more effective replacement levels are required. Therefore, the objective of this study was to evaluate how using alternative diets with low content of marine resources can affect growth performance, and muscle lipid class and fatty acid composition in farmed Atlantic salmon. This study improves the understanding of the nutrition requirements of Atlantic salmon under various dietary conditions, and provides an insight into the potential performance of future aquafeed alternatives.

## Materials and methods

### Experimental diets

Seven different diets were produced by EWOS Innovation AS in Norway. The diets were formulated using different levels of fish meal, fish oil, animal by-products, vegetable oil, and vegetable protein, yielding different levels of DHA+EPA. The seven diets were characterized according to the most critical component used and designated as marine, with high levels of fish meal and fish oil; medium marine, containing medium levels of fish meal and fish oil; animal by-product, composed of a high proportion of animal by-products; vegetable protein, including a high level of vegetable protein; ω3LC0, which contained approximately 0% long-chain ω3 fatty acids (LC ω3 FAs); ω3LC1, with 1% of LC ω3 FAs; and ω3LC1.41, with 1.4% of LC ω3 FAs (Table 1).

**Table 1:** Experimental diet ingredients.

Ingredients^1^	Marine	Medium marine	Animal by-product	Vegetable protein	ω3LC0	ω3LC1	ω3LC1.41
**Fish meal^2^ (%)**	**35**	**15**	5	5	5	5	5
**Animal by-products^3^ (%)**	15	26	**33**	10	22	21	22
**Corn gluten**	1.4	3.0	3.4	**7.0**	5.1	5.0	5.0
**Soy protein concentrate**	7	15	17	**35**	25	25	25
**Wheat gluten meal**	0.9	2.0	2.3	**4.7**	3.4	3.3	3.3
**Fish oil (%)**	**12**	**6**	5	5	**0**	5	7
**Vegetable oil^4^ (%)**	6	13	14	17	27	22	20
**Raw wheat**	23	20	18	13	10	10	10
**Premix^5^**	0.5	0.7	1.1	3.0	2.4	2.3	2.4
**Digestible energy (MJ Kg^-1^)**	20	20	20	20	21	21	21
**Digestible protein (g Kg^-1^)**	375	375	375	375	360	360	360
**EPA+DHA (%)**	2.91	1.41	1.00	1.00	**0.09**	**1.00**	**1.41**

Boldfaced numbers indicate key components of each diet

^1^ The ingredients were all sourced from EWOS stocks.

^2^ Anchovy fish meal

^3^ Poultry, feather, and blood meal. For confidentiality, the proportions of the Animal by-products in the diets are not provided.

^4^ Rapeseed oil

^5^ Premix contains vitamins, trace elements and inorganic phosphorus. The detailed composition of the premix is proprietary information to EWOS.

### Experimental fish and feeding

Atlantic salmon (*Salmo salar*) smolts were obtained from Northern Harvest Sea Farms in Stephenville, NL, Canada. Fish were tagged with passive transponders (PIT) and maintained at 11±1°C in a flow-through seawater system under a 12-h light photoperiod in the Dr. Joe Brown Aquatic Research Building in St. John’s, Newfoundland, Canada. Fish (1148 at 139–232 g each) were distributed randomly in 28 tanks (620 L, 4 tanks per diet). There were 41 fish per tank until day 0, when one fish/tank was sampled in order to have a random selection of fish representing every tank at week 0. Fish were hand fed 5 mm experimental pellets to satiation twice a day for 14 weeks. Feed consumption was measured weekly and fish weight and fork length were recorded at the beginning and end of the experiment.

### Tissue sampling

Ten fish were randomly sampled at week 0, followed by 5 fish per tank at week 7 and 14. Fish were euthanized with an overdose of MS-222 (400 mg L^-1^, Syndel Laboratories, Vancouver, BC, Canada), then fork length and weight were measured. Gut and liver were removed, weighed, sampled, and placed on aluminum weigh boats. Norwegian quality cuts (NQC) (area from directly behind the dorsal fin to the anus) were removed from the fish body and weighed. With the dorsal fin cut to the right, the skin was cut along the dorsal side, peeled back to expose the skeletal white muscle (hereafter referred to as muscle), and a strip (approximately 0.5 g) of muscle was removed and placed into lipid clean test tubes (rinsed with methanol and chloroform three times each) for lipid analysis. The tubes had been weighed after ashing at 450°C for 8 hrs, and the Teflon lined caps were rinsed three times with methanol and chloroform. Vials were kept on ice during sampling. After sampling, 2 ml of chloroform was poured on the tissues in the tubes and the remaining space was filled with nitrogen. The tubes were then sealed and stored at -20°C. All procedures, including handling, treatment, euthanasia, and dissection were performed according to the guidelines from the Canadian Council of Animal Care (approved Memorial University Institutional Animal Care Protocol 14e71-MR).

### Lipid extraction and lipid class determination

Samples were ground in methanol:chloroform:water and extracts were evaporated to volume under a gentle stream of nitrogen before sealing and storing at -20°C [22]. Lipid class composition was determined through a three-step development method using thin-layer chromatography–flame ionization detection [23]. Lipid extracts and standards were spotted on Chromarods and the rods were developed and then scanned in an Iatroscan. Data were collected using PeakSimple software.

### Fatty acid methyl ester (FAME) derivatization

Fifty microlitres of lipid extract was transferred into 15 ml lipid clean vials and concentrated to dryness. Methylene chloride (1.5 ml) and 3 ml of Hilditch reagent (1.5 H_2_SO_4_: 98.5 anhydrous methanol) were added, then vials were vortexed and sonicated for 4 min. They were filled with nitrogen, capped and heated at 100°C for 1 hr. Saturated sodium bicarbonate solution (0.5 ml) and 1.5 ml hexane were added to the samples which were vortexed, followed by removing the upper, organic layer. The vials were then dried and refilled with hexane to approximately 0.5 ml. They were filled with nitrogen, capped, sealed with Teflon tape, and finally sonicated for another 4 min to re-suspend the fatty acids.

### Lipid oxidation

Lipid oxidation was measured according to the thiobarbituric acid reactive substance (TBARS) method [24]. To measure lipid oxidation, 1 gram of muscle sample (two replicates per fish) was weighed and transferred into centrifuge tubes (plus 1 blank). The sample was homogenized with 5 ml of 5% (w/v) trichloroacetic acid (TCA) by polytron. Samples were centrifuged at 3000 rpm for 10 min and the supernatant (top layer) was filtered through a 0.45 μm pore syringe filter. Five ml of 0.08 M thiobarbituric acid (TBA) and 2.5 ml of TCA were added and heated in a boiling water bath at 94±1°C for 45 min then cooled to room temperature. Finally, the absorbance was measured at 532 nm using a UV-spectrophotometer. Thiobarbituric acid reactive substances (TBARS) values were calculated using a standard curve. Standard curves were prepared using 1,1,3,3-tetramethoxypropane as a precursor of malondialdehyde (MDA; 0–10 ppm).

### Statistics

To ensure representative fish were sampled for analysis, only fish with weight gains within the range of twice the standard deviation from the overall tank weight gain means were considered. Correlation and regression analyses were conducted using Minitab version 17 to compare diet ingredients, lipid and fatty acid composition of muscle and diet, and growth characteristics. For statistical analysis of growth data, lipid class, and fatty acid data, nested general linear models were combined with Tukey pairwise comparisons using Minitab to determine the difference between tanks and diets. The normality of residuals was evaluated with the Anderson-Darling normality test. If the test failed (p<0.05), a one-way analysis of variance (ANOVA) on ranks was performed in SigmaPlot version 13.

## Results

### Experimental diet composition

Moisture levels in the feeds ranged from 5.0 to 7.5% and diets contained 92.5 to 95.0% dry matter. There were significant differences in proximate composition data among the seven diets; however, these differences were small (Table 2). While there was no significant difference in the nitrogen proportion among the seven diets, the carbon was statistically different between the marine and ω3LC1.41 diets, which contained the lowest and highest levels of C, respectively (Table 2). The major lipid class was triacylglycerol ranging from 92 mg g^-1^ in the animal by-product diet to 173 mg g^-1^ in the ω3LC0 diet (Table 3). All diets had the same concentrations of 16:0, ƩSFA, and ƩPUFA as well as the same ratios of PUFA/SFA and DHA/EPA. The three diets with 5% fish meal and ~22% animal by-products, ω3LC0, ω3LC1, and ω3LC1.41 had statistically the same content of 18:1ω9, 18:2ω6, 18:3ω3, and 20:4ω6. The 20:5ω3 and 22:6ω3 contents were significantly lower in the ω3LC0 diet than in the marine, medium marine and ω3LC1.41 diets (Table 3).

**Table 2:** Proximate and elemental composition of experimental diets.

	Marine n = 3	Medium marine n = 3	Animal by-product n = 3	Vegetable protein n = 3	ω3LC0 n = 3	ω3LC1 n = 3	ω3LC1.41 n = 3
**Moisture** **(% wet weight)**	7.5±0.1^a^	5.2±0.6^cd^	5.0±0.2^cd^	6.9±0.0^a^	5.0±0.4^d^	5.9±0.3^bc^	6.7±0.4^ab^
**Dry matter (% wet weight)**	92.5±0.1^d^	94.8±0.6^ab^	95.0±0.2^ab^	93.1±0.0^d^	95.0±0.4^a^	94.1±0.3^bc^	93.3±0.4^cd^
**Organic matter (% dry weight)**	91.2±0.4^c^	92.7±0.2^b^	93.4±0.1^a^	93.6±0.2^a^	93.5±0.2^a^	93.5±0.2^a^	93.7±0.3^a^
**Organic matter (% wet weight)**	84.8±0.5^c^	87.9±0.7^ab^	88.8±0.3^a^	87.1±0.2^b^	88.8±0.5^a^	88.0±0.4^ab^	87.4±0.2^b^
**Ash (% dry weight)**	8.8±0.4^a^	7.3±0.2^b^	6.6±0.1^c^	6.4±0.2^c^	6.5±0.2^c^	6.5±0.2^c^	6.3±0.3^c^
**Ash (% wet weight)**	8.1±0.4^a^	6.9±0.2^b^	6.2±0.1^c^	6.0±0.2^c^	6.2±0.2^c^	6.1±0.2^c^	5.9±0.3^c^
**C^1^**	46.4±0.5^b^	46.9±0.6^ab^	48.3±0.4^ab^	46.8±2.3^ab^	48.8±0.4^ab^	48.4±0.5^ab^	49.2±0.5^a^
**N^2^**	6.8±0.1	7.4±0.3	7.3±0.1	7.1±0.6	7.2±0.1	7.3±0.1	7.3±0.1
**C/N**	6.8±0.1^a^	6.3±0.2^b^	6.6±0.1^ab^	6.6±0.2^ab^	6.7±0.1^ab^	6.6±0.1^ab^	6.7±0.1^ab^

Data are mean ± standard deviation for n replicates; different letters indicate significant differences

^1^ Carbon (% dry weight)

^2^ Nitrogen (% dry weight)

**Table 3:** Lipid class and fatty acid composition of experimental diets, as fed (mg g^-1^ wet weight).

	Marine n = 6	Medium marine n = 6	Animal by-product n = 6–9	Vegetable protein n = 6	ω3LC0 n = 6–9	ω3LC1 n = 9	ω3LC1.41 n = 6–9
***Lipid classes*** ***(mg g*^*-1*^*wet weight)***							
Total lipid	161.1±64.5^ab^	173.4±46.7^ab^	140.1±39.6^b^	159.3±27.6^ab^	229.2±59.2^a^	203.5±25.6^ab^	208.7±38.7^a^
Triacylglycerol	93.3±45.2^b^	96.1±28.1^b^	92.1±40.6^b^	115.4±27.3^ab^	173.2±55.0^a^	149.6±27.7^ab^	147.8±34.9^ab^
***Fatty acid*** ***(mg g*^*-1*^*wet weight)***							
16:0	14.1±6.1	13.5±4.0	9.9±3.8	10.3±2.5	10.8±1.8	13.0±3.0	13.5±2.1
18:1ω9	30.7 ± 13.2^e^	49.6 ± 13.8^cde^	44.6 ± 17.0^de^	56.6 ± 12.0^bcd^	88.4 ± 15.4^a^	77.4 ± 17.0^ab^	71.0± 11.5^abc^
18:2ω6	12.3 ± 5.3^d^	20.1 ± 5.7^bcd^	17.7± 6.8^cd^	22.8 ± 5.1^abc^	32.0 ± 5.8^a^	29.4 ± 6.7^ab^	27.1 ± 4.3^abc^
18:3ω3	4.8 ± 2.1^e^	7.5 ± 2.1^cde^	6.6 ± 2.5^de^	8.8 ± 1.9^bcd^	12.7 ± 2.5^a^	11.9 ± 2.7^ab^	10.9 ± 1.8^abc^
20:4ω6	0.5± 0.2^a^	0.5 ± 0.1^ab^	0.3 ± 0.1^abc^	0.2± 0.1^c^	0.2 ± 0.1^c^	0.3 ± 0.9^bc^	0.4 ± 0.1^abc^
20:5ω3	8.3 ± 3.6^a^	5.1 ± 1.5^a^	2.6 ± 1.0 ^ab^	3.1± 0.8^ab^	0.6 ± 0.1^b^	3.3 ± 0.8 ^ab^	4.3 ± 0.7^a^
22:6ω3	7.7 ± 3.4 ^a^	4.7 ± 1.4 ^a^	2.4 ± 0.8 ^ab^	3.0± 1.0 ^ab^	0.7 ± 0.1 ^b^	3.1 ± 0.8 ^ab^	3.9 ± 0.7 ^a^
Ʃ SFA^1^	24.1 ± 10.3	24.6 ± 7.2	19.9 ± 7.8	20.7 ± 4.7	22.8 ± 3.8	25.4 ± 5.8	22.9 ± 3.6
Ʃ MUFA^2^	63.9 ± 27.4^bc^	74.5 ± 20.6^abc^	60.7 ± 23.2^c^	75.3 ± 16.0^abc^	99.7 ± 17.1^a^	99.5 ± 21.8^a^	97.0 ± 15.5 ^ab^
Ʃ PUFA^3^	43.0 ± 18.8	43.6 ± 12.5	32.8 ± 12.4	41.6 ± 9.6	47.5 ± 8.9	52.6 ± 11.9	51.6 ± 8.3
*P/S^4^	1.8 ± 0.1	1.8 ± 0.1	1.7 ± 0.1	2.0 ± 0.0	2.0 ± 0.20	2.0 ± 0.1	2.2 ± 0.0
Ʃ ω3^5^	26.4 ± 11.6 ^a^	20.5 ± 6.0 ^ab^	13.4 ± 5.0^b^	17.0 ± 4.1^ab^	14.6 ± 3.0^b^	21.1 ± 5.0 ^ab^	22.0 ± 3.6^ab^
*DHA/EPA	0.9 ± 0.0	0.9 ± 0.0	0.9 ± 0.0	1.0 ± 0.1	1.2 ± 0.1	1.0 ± 0.0	0.9 ± 0.0

* Unitless ratios

^1^ Saturated fatty acids, including 14:0, Trimethyltridecanoic acid (TMTD), 15:0, pristanic?, 16:0, phytanic?, 17:0, 18:0, 19:00, 20:0, 21:00, 22:0, 23:0, 24:0

^2^ Monounsaturated fatty acids, including 14:1, 15:1, 16:1ω11?, 16:1ω9?, 16:1ω7, 16:1ω5, 17:1,1 8:1ω11?, 18:1ω9, 18:1ω7, 18:1ω6?, 18:1ω5?, 20:1ω11?, 20:1ω9, 20:1ω7?, 22:1ω11(13), 22:1ω9, 22:1ω7, 24:1

^3^ Polyunsaturated fatty acids, including 16:2ω4, 16:3ω4?, 16:3ω3?, 16:4ω3?, 16:4ω1, 18:2a, 18:2b, 18:2ω6, 18:2ω4, 18:3ω6, 18:3ω4, 18:3ω3, 18:4ω3, 18:4ω1?, 18:5ω3, 20:2a?, 20:2b?, 20:2ω6, 20:3ω6, 20:4ω6, 20:3ω3, 20:4ω3, 20:5ω3, 22:2NIMDa?, 22:2NIMDb?, 21:5ω3?, 22:4ω6?, 22:5ω6, 22:4ω3?, 22:5ω3, 22:6ω3

^4^ Polyunsaturated/saturated fatty acids

^5^ Ʃ ω3, including 16:3ω3?, 16:4ω3?, 18:3ω3, 18:4ω3, 18:5ω3, 20:3ω3, 20:4ω3, 20:5ω3, 21:5ω3?, 22:4ω3?, 22:5ω3, 22:6ω3

Data are mean ± standard deviation for n replicates; different letters indicate significant differences

### Growth performance

After 14 weeks of feeding, final weights were lowest when using the diet with the lowest fish meal and fish oil content (ω3LC0), followed by the diet containing the highest level of animal by-products. Fish fed diets ω3LC0, ω3LC1, and ω3LC1.41 had the lowest hepatosomatic index (HSI) and fish fed the marine diet had the highest HSI (Table 4). The lowest specific growth rate (SGR) was obtained with the ω3LC0 and animal by-product diets (Table 4). The condition factor (CF) was not influenced due to relatively similar growth in body weight and length, which shows that the diets were well designed.

**Table 4:** Salmon growth data—week 14.

Diet	Marine n = 134	Medium marine n = 134	Animal by-products n = 138	Vegetable protein n = 137	ω3LC0 n = 135	ω3LC1 n = 140	ω3LC1.41 n = 137
**Initial Weight (g)**	176.8 ±29.4	179.3 ± 30.3	179.2± 28.8	177.2± 29.8	176.6± 63.7	179.3± 30.1	178.1± 27.5
**Final Weight (g)**	342.5± 89.5^a^	325.5± 74.1^ab^	316.3± 63.6^b^	332.9± 79.2^ab^	309.3± 63.7^b^	339.7± 73.5^ab^	341.9± 68.7^ab^
**Weight Gain (g)**	165.6 ± 77.5^a^	146.2 ± 54.7^b^	137.1 ± 47.5^b^	155.7 ± 64.6^ab^	132.7 ± 48.6^b^	160.4 ± 59.7^ab^	163.8 ± 54.6^ab^
**Initial Length (cm)**	24.9± 1.3	25.0± 1.3	25.0± 1.4	25.0±1.4	24.9±1.2	25.0± 1.3	25.0± 1.3
**Final Length (cm)**	30.4± 2.4^ab^	30.1± 2.2^ab^	30.0± 2.1^ab^	30.2± 2.5^ab^	26.6± 2.0^a^	30.6± 2.0^b^	30.6± 2.0^ab^
**Length Gain (cm)**	5.5 ± 1.9^a^	5.2 ± 1.4^ab^	5.0 ± 1.3^b^	5.2 ± 2.0^ab^	4.9 ± 1.5^b^	5.6 ± 1.6^a^	5.6 ± 1.5^a^
NQC* **(g)**	59.8± 17.8	57.5± 14.6	49.6± 10.8	58.0± 12.8	54.4± 10.6	58.6± 12.0	58.6± 11.6
**SGR^1^ (% day^-1^)**	0.65± 0.26^a^	0.59± 0.18^bc^	0.57± 0.14^c^	0.62± 0.23^ab^	0.56± 0.18^c^	0.64± 0.21^ab^	0.66± 0.17^a^
**CF^2^ (g cm^-1^)**	1.19± 0.14	1.17± 0.11	1.16± 0.10	1.20± 0.28	1.15± 0.09	1.17± 0.11	1.18± 0.10
**VSI^3^ (%)**	11.4± 1.4	10.6± 1.8	10.7± 1.4	11.1± 1.7	10.9± 1.5	11.3± 1.7	11.1± 1.3
**HSI^4^ (%)**	1.49± 0.40^a^	1.28± 0.33^ab^	1.29± 0.38^ab^	1.19± 0.25^b^	1.09± 0.22^b^	1.14± 0.22^b^	1.10± 0.18^b^
**AFI^5^ (g fish^-1^)**	178.7± 25.2	158.4± 7.0	146.6± 5.5	167.4± 14.4	145.4± 16.7	159.0± 11.2	161.5± 19.7
**FCR^6^**	1.09± 0.06	1.09± 0.06	1.07± 0.03	1.08± 0.08	1.10± 0.05	0.99± 0.03	0.99± 0.02
**VFI^7^**	0.25±0.02^a^	0.24±0.01^ab^	0.22±0.00^ab^	0.25±0.01^ab^	0.22±0.02^b^	0.23±0.01^ab^	0.23±0.02^ab^

* Norwegian quality cut; the area from directly behind the dorsal fin to the anus, n = 18–20

^1^ Specific growth rate = 100 × [ln(final body weight) − ln(initial body weight)]/days

^2^ Condition factor = body mass/length^3^

^3^ Viscerosomatic index = 100 × (viscera mass/body mass), n = 29–32

^4^ Hepatosomatic index = 100 × (liver mass/body mass), n = 29–32

^5^ Apparent feed intake = feed consumption/number of fish per tank, n = 4

^6^ Feed conversion ratio = feed consumption/weight gain, n = 4

^7^ Voluntary feed intake = feed consumption/average body weight/day, n = 4

Data are mean ± standard deviation for n replicates; different letters indicate significant differences

### Elemental composition

Elemental compositions were measured at week 7 and 14 in muscle tissue (Norwegian quality cut). The C/N ratio and N proportion in muscle tissues differ significantly among the seven diets after week 7. However, there was no significant difference in the C/N ratio and C proportion of muscle tissue at week 14. Also, no significant differences in muscle N proportion were observed when feeding the seven diets at week 14, except between the marine (lowest N) and animal by-products (highest N) diets (Table 5).

**Table 5:** Elemental composition of muscle tissues at week 14.

Elemental Composition	Marine n = 20	Medium marine n = 20	Animal by-product n = 20	Vegetable protein n = 20	ω3LC0 n = 20	ω3LC1 n = 20	ω3LC1.41 n = 20
**C^1^**	49.3±3.3	47.9±3.4	49.6±3.8	49.3±1.8	47.8±5.4	49.3±1.6	48.9±3.8
**N^2^**	13.4±0.7^b^	13.6±0.7^ab^	14.9±2.7^a^	13.5±1.3^ab^	13.6±1.5^ab^	13.9±0.6^ab^	13.4±1.1^ab^
**C/N**	3.7±0.4	3.5±0.2	3.4±0.3	3.7±0.5	3.5±0.2	3.6±0.2	3.7±0.4

Data are mean ± standard deviation for 20 replicates; different letters indicate significant differences

^1^ Carbon (% dry weight)

^2^ Nitrogen (% dry weight)

### Muscle lipid class composition and fatty acid composition

There was no significant difference in total lipids among the different treatments but muscle triacylglycerol was lowest with the animal by-product diet and highest with the ω3LC1.41 diet (Table 6). Fish fed the marine and animal by-products diets showed the highest and lowest level of sterol in muscle tissues respectively (Table 6). Diet ω3LC0 resulted in significantly higher contents of 20:4ω6 than medium marine diet. Animal by-products, ω3LC0, and ω3LC1.41 diets led to the highest DHA/EPA ratio, while the marine diet resulted in the lowest ratio (Table 7).

**Table 6:** Lipid classes of muscle tissue before and after 14 weeks of feeding (mg g^-1^ wet weight).

Lipid classes (mg g^-1^ wet weight)	Initial n = 10	Marine n = 16–19	Medium marine n = 16–19	Animal by-products n = 16–19	Vegetable protein n = 16–19	ω3LC0 n = 16–19	ω3LC1 n = 16–19	ω3LC1.41 n = 16–19
**Total lipid**	15.2±7.6	17.8±14.0	9.3±5.2	8.6±4.7	13.7±5.5	10.6±6.0	17.2±12.3	18.1±15.3
**Triacylglycerol**	7.2±4.8	8.6±7.7^ab^	5.6±4.5^ab^	3.4±2.1^b^	7.9±4.9^ab^	5.1±3.4^ab^	8.3±6.4^ab^	9.2±6.7^a^
**Sterol**	0.26±0.17	0.87±0.77^a^	0.17±0.09^ab^	0.20±0.33^b^	0.23±0.04^a^	0.26±0.16^a^	0.29±0.27^ab^	0.28±0.18^a^
**Phospholipid**	5.0±2.4	2.8±0.8^b^	2.9±1.3^b^	3.9±1.5^ab^	3.8±0.6^ab^	4.2±2.8^ab^	4.4±2.5^ab^	5.2±3.2^a^

Underlined data represent values that are statistically different from week 0; different letters indicate significant differences among diets

Data are mean ± standard deviation for n replicates

**Table 7:** Fatty acid composition of muscle tissue before and after 14 weeks of feeding (mg g^-1^ wet weight).

Fatty acid (mg g^-1^ wet weight)	Initial n = 10	Marine n = 12–19	Medium marine n = 12–19	Animal by-product n = 12–19	Vegetable protein n = 12–19	ω3LC0 n = 12–19	ω3LC1 n = 12–19	ω3LC1.41 n = 12–19
**14:0**	0.38±0.24	0.5±0.45^a^	0.16±0.14^ab^	0.09±0.06^ab^	0.18±0.10^a^	0.07±0.05^b^	0.21±0.14^a^	0.25±0.24^a^
**16:0**	1.90±0.91	2.18±1.61	1.03±0.58	0.94±0.41	1.36±0.51	0.94±0.65	1.60±0.86	1.59±1.11
**16:1ω7**	0.65±0.41	0.64±0.63	0.22±0.19	0.13±0.09	0.24±0.15	0.11±0.09	0.30±0.22	1.20±0.39
**18:0**	0.46±0.24	0.52±0.43	0.25±0.15	0.22±0.11	0.34±0.15	0.27±0.16	0.42±0.27	0.42±0.33
**18:1ω7**	0.35±0.20	0.40±0.33^ab^	0.20±0.13^ab^	0.17±0.08^b^	0.29±0.14^ab^	0.23±0.15^ab^	0.39±0.25^a^	0.37±0.29^ab^
**18:1ω9**	2.29±1.36	3.88±3.72^ab^	2.13±1.63^ab^	1.70±1.08^b^	3.56±2.08^ab^	2.95±2.09^ab^	5.02±3.56^a^	4.55±3.97^a^
**18:2ω6**	0.98±0.60	1.25±1.14^ab^	0.74±0.56^ab^	0.56±0.37^b^	1.19±0.66^ab^	0.89±0.62^ab^	1.63±1.15^a^	1.52±1.26^ab^
**18:3ω3**	0.12±0.07	0.37±0.32^ab^	0.20±0.14^ab^	0.15±0.08^b^	0.32±0.16^a^	0.23±0.15^ab^	0.43±0.32^a^	0.41±0.29^a^
**18:4ω3**	0.11±0.07	0.27±0.25	0.11±0.09	0.09±0.05	0.18±0.11	0.17±0.10	0.22±0.17	0.23±0.21
**20:1ω9**	0.23±0.15	0.84±0.80^ab^	0.25±0.20^ab^	0.16±0.10^b^	0.33±0.19^a^	0.12±0.09^b^	0.39±0.34^ab^	0.47±0.41^a^
**20:4ω6**	0.14±0.06	0.12±0.06^ab^	0.07±0.03^b^	0.09±0.04^ab^	0.11±0.03^ab^	0.14±0.07^a^	0.12±0.05^ab^	0.10±0.05^ab^
**20:5ω3**	0.65±0.26	0.67±0.42^a^	0.29±0.12^b^	0.28±0.11^b^	0.34±0.09^ab^	0.24±0.13^b^	0.38±0.16^ab^	0.37±0.20^ab^
**22:1ω11(13)**	0.19±0.14	1.97±0.78^a^	2.09±0.17^a^	2.18±0.08^ab^	2.09±0.14^a^	2.20±0.03^b^	2.15±0.22^a^	2.23±0.21^a^
**22:5ω3**	0.23±0.11	0.25±0.19	0.09±0.05	0.08±0.04	0.11±0.04	0.08±0.04	0.12±0.06	0.11±0.08
**22:6ω3**	2.24±0.80	2.48±1.20^a^	1.26±0.41^bd^	1.41±0.53^bd^	1.60±0.38^abd^	1.25±0.70^cd^	1.80±0.76^abd^	1.90±0.77^ab^
**Ʃ SFA^1^**	2.84±1.45	3.39±2.64	1.54±0.92	1.37±0.62	2.08±0.83	1.47±0.96	2.48±1.40	2.56±2.15
**Ʃ MUFA^2^**	3.91±2.36	6.98±6.62	3.16±2.42	2.37±1.49	4.85±2.79	3.58±2.52	6.66±4.74	6.30±5.36
**Ʃ PUFA^3^**	4.99±2.24	6.16±4.15	3.06±1.54	2.93±1.17	4.26±1.50	3.34±1.89	5.28±2.91	5.20±3.16
*P/S^4^	1.81±0.12	1.97±0.25	2.09±0.23	2.18±0.21	2.09±0.17	2.20±0.10	2.15±0.08	2.23±0.38
**Ʃ ω3^5^**	3.47±1.37	4.28±2.54^a^	2.03±0.83^b^	2.07±0.76^b^	2.65±0.73^ab^	2.02±1.10^b^	3.08±1.49^ab^	3.21±1.59^ab^
*DHA/EPA	3.54±0.31	4.03±0.77^b^	4.50±0.71^ab^	5.05±0.64^a^	4.77±0.70^ab^	5.22±0.86^a^	4.82±0.57^ab^	4.94±0.90^a^

* Unitless ratios

^1^ Saturated fatty acids, including 14:0, Trimethyltridecanoic acid (TMTD), 15:0, pristanic?, 16:0, phytanic?, 17:0, 18:0, 19:00, 20:0, 21:00, 22:0, 23:0, 24:0

^2^ Monounsaturated fatty acids, including 14:1, 15:1, 16:1ω11?, 16:1ω9?, 16:1ω7, 16:1ω5, 17:1,1 8:1ω11?, 18:1ω9, 18:1ω7, 18:1ω6?, 18:1ω5?, 20:1ω11?, 20:1ω9, 20:1ω7?, 22:1ω11(13), 22:1ω9, 22:1ω7, 24:1

^3^ Polyunsaturated fatty acids, including 16:2ω4, 16:3ω4?, 16:3ω3?, 16:4ω3?, 16:4ω1, 18:2a, 18:2b, 18:2ω6, 18:2ω4, 18:3ω6, 18:3ω4, 18:3ω3, 18:4ω3, 18:4ω1?, 18:5ω3, 20:2a?, 20:2b?, 20:2ω6, 20:3ω6, 20:4ω6, 20:3ω3, 20:4ω3, 20:5ω3, 22:2NIMDa?, 22:2NIMDb?, 21:5ω3?, 22:4ω6?, 22:5ω6, 22:4ω3?, 22:5ω3, 22:6ω3

^4^ Polyunsaturated/saturated fatty acids

^5^ Ʃ ω3, including 16:3ω3?, 16:4ω3?, 18:3ω3, 18:4ω3, 18:5ω3, 20:3ω3, 20:4ω3, 20:5ω3, 21:5ω3?, 22:4ω3?, 22:5ω3, 22:6ω3

Underlined data represent values that are statistically different from week 0; different letters indicate significant differences among diets

Data are mean ± standard deviation for n replicates

### Correlation analysis

Apparent feed intake, final weight and weight gain showed a significant positive correlation with the amount of fish oil in the diet, as did HSI; however, SGR, final length, NQC, VSI and CF did not correlate significantly with fish oil level. Ʃω3 in the diet and muscle had positive correlations with growth performance characteristics (r = 0.82–0.87, S1 Table) but there were significant positive correlations between HSI and fish meal, fish oil, and EPA+DHA (r = 0.77–0.88). By contrast, there was an inverse correlation between HSI and vegetable oil content in the diet (-0.942, P<0.01), which is why the diet with the highest inclusion of vegetable oil (ω3LC0) resulted in the lowest HSI (S1 Fig, Table 4). Animal by-products inclusion showed a negative correlation with condition factor (CF, S1 Table), while there was a positive linear relationship between the content of 22:6ω3 in diet and muscle dietary essential fatty acids and muscle fatty acids for 20:5ω3 and 22:6ω3 (P<0.05, R^2^ = 75.8% and P<0.05, R^2^ = 60%, respectively, S2 Table and S2 Fig).

### Lipid oxidation

Lipid oxidation was also studied to measure the extent it could be affected by muscle tissue lipids, as high content of PUFA could make them susceptible to oxidation. Oxidation results were represented by TBARS values for marine, animal by-product, and ω3LC1.41 diets with mean values of 1.15±0.61, 1.52±0.65, and 1.01±0.59 mg MDA kg^-1^ of sample (mg of malondialdehyde equivalents per kg of muscle sample), respectively.

## Discussion

While the global consumption of marine ingredients by aquaculture continues to increase, their production has not changed significantly [2] and has even declined slightly in recent years [25]. Consequently, this study evaluated the effects of replacing fish meal and fish oil in aquafeeds with soy protein concentrate, poultry, feather, and blood meal, and with rapeseed oil.

Cultured fish need lipid, protein, energy, vitamins and minerals in their diet for growth, reproduction, and other normal physiological functions. Lipid contributes to the structure of biomembranes [26] and has a critical role in providing energy for animal tissues and is the source of essential fatty acids. The poor performance of fish fed the ω3LC0 diet is undoubtedly associated with the complete lack of fish oil in this diet [27]. Fish meal contains valuable eicosapentaenoic acid (EPA, 20:5ω3) and docosahexaenoic acid (DHA, 22:6ω3) and a relatively high content of fish meal in the diet reduces the possibility of having low levels of essential fatty acids [12]. The content of fish meal in the diet of Atlantic salmon was reduced to 25% in [10] with no significant change in growth and feed conversion. However, the present study further reduced the fish meal content to as low as 5%.

The ω3LC0 diet also resulted in the lowest HSI which is contrary to the results of other studies on salmonids (e.g. [28], [29]), in which HSI was statistically unaffected by the increase of vegetable oil content in the diet. However, in those studies the maximum vegetable oil content was 20%, whereas here the ω3LC0 diet had the highest inclusion of vegetable oil at 27%.

Correlation analysis showed that growth performance characteristics are positively correlated with total concentrations of ω3 fatty acids in the diets, but interestingly there was no significant correlation with individual ω3 fatty acids in the diets suggesting that an improvement in growth performance may be obtained irrespective of ω3 fatty acid chain-length or degree of unsaturation. In turn this indicates an interchangeability among these fatty acids in terms of function or biochemically through modification of chemical structures, e.g. chain elongation and desaturation.

With the exception of the animal by-product diet, diets containing ≥5% fish meal and ≥5% fish oil gave the same final weight, and length gain, probably because the minimum level of essential fatty acids was provided [30]. However, as with the ω3LC0 diet, the animal by-product diet resulted in significantly lower growth despite containing 5% fish meal and 5% fish oil, which may relate to lipid oxidation. While muscle in fish fed the animal by-product diet was expected to be less exposed to lipid oxidation due to the lower content of PUFAs (Table 7), the TBARS results revealed that these fish muscle samples were oxidized significantly more than the ones fed ω3LC1.41. This may reflect the types of materials used for animal by-products and how they were handled. Nonetheless, the oxidation results suggested that lipid rancidity was not high in any of the three diet treatments studied, as the TBARS values were significantly lower than the results of other studies on salmon (e.g. [31]). A TBARS value of about 16.5 mg MDA kg^-1^ was obtained in [31] after 6 days, while the TBARS values in this study did not exceed 3.13 mg MDA kg^-1^ (mean of 1.52 mg MDA kg^-1^). Also, lower levels of animal by-products may be well tolerated. It was shown in [32] that 7% inclusion of porcine blood meal had no negative effects on salmon performance compared with fish meal based control diets. The animal by-product diet also resulted in the lowest concentrations of muscle triacylglycerols which is consistent with the down-regulation of fatty acid synthesis in the livers of these fish [33].

The vegetable protein and ω3LC1 diets were remarkable in giving the same results for final weight, weight gain, final length, length gain, and SGR as when the marine diet was used, despite having less than half the amount of fish oil in the marine diet and as little as 1/7 of the fish meal. Even though other studies reported poor [34] or the same ([35], [36]) growth performance when fish meal was partially replaced with vegetable proteins, with about the same or even more fish oil content than the control diet, the vegetable protein diet in this study contained much lower levels of fish meal and fish oil content than other studies and still resulted in the same growth performance as the marine diet. In addition, the vegetable protein diet was found to increase the immune response of these fish [37].

For fish fed diets containing 5–7% fish oil and 5–15% fish meal, there was no significant difference in the concentrations of DHA nor EPA in the muscle. EPA and DHA have anti-inflammatory effects, which make fish resistant to diseases [1]. As expected, DHA and EPA was lowest with the ω3LC0 diet, but ARA was highest (Table 7). This despite the concentration of ARA being lowest in this diet along with the vegetable protein diet. This diet contains a high inclusion of rapeseed oil which has a high level of 18:2ω6, so conversion to 20:4ω6 [38] in muscle tissues is strongly indicated.

While five of the treatments gave no significant difference in muscle DHA and EPA concentrations the two with the lowest and highest levels of fish oil did give significant differences, as expected ([39], [15], [40]). This resulted in significant regressions between 20:5ω3 and 22:6ω3 in diets and muscle tissues. The slope of the regression line for dietary content of 20:5ω3 and 22:6ω3 against their contents in muscle tissue indicates the extent of this dependency. The higher slope for 22:6ω3 (0.30 mg g^-1^ wet weight) compared to 20:5ω3 (0.08 mg g^-1^ wet weight) showed that 22:6ω3 was deposited four times more than 20:5ω3 in muscle tissue. A similar result was observed when fish oil was replaced with crude palm oil in Atlantic salmon diets [17], where 22:6ω3 (slope = 0.81) was more deposited than 20:5ω3 (slope = 0.58) in muscle tissues when proportions (% total fatty acids) were compared. Similar to other studies (e.g. [13], [39], [28], [9], [41], [42]), the concentration of long chain MUFAs (20:1ω9 and 22:1ω11(13)) in the muscle tissues were highest for fish fed marine based diets (2.81 mg g wet weight^-1^ total). However, unlike the previous studies in which the replacement of marine based ingredients generally resulted in lower proportions of long chain MUFAs, the use of other diets led to statistically the same concentrations of 20:1ω9 and 22:1ω11(13) in muscle as when marine based diets were fed, with a couple of exceptions (Table 7). The difference in the results here to those previously probably relates to the determination of concentrations *versus* proportions.

The fatty acid compositions of muscle tissues (Table 7) show that the sum of EPA and DHA levels ranges from 1.49 to 3.15 mg g^-1^ wet weight. According to the Canada’s Food Guide [43], one serving of cooked fish is 75 g. Therefore, a serving of Atlantic salmon fed the seven dietary treatments includes EPA+DHA levels ranging from 112 to 236 mg which would be less than the daily requirement (250 mg) recommended by the World Health Organization [44], even for fish fed the marine diet. This is consistent with the decreasing trend in the content of EPA+DHA in commercially farmed salmon overtime [45]. Therefore, to meet the recommended daily EPA+DHA value, one would need to eat more than one serving of cooked fish.

## Conclusion

This study evaluated the effects of minimizing marine resource utilization in diets of farmed Atlantic salmon on growth and muscle lipid composition. By replacing fish meal and fish oil in aquafeeds with animal by-products and rapeseed oil at levels used in this study (33 and 27%, respectively), marine resource utilization was reduced to 10% or less; however, this affected growth, lipid class, and fatty acid composition of muscle tissue. When the content of animal by-products and vegetable oil in the diet did not exceed 26% and 22%, respectively most growth, lipid class, and fatty acid parameters remained unaffected.

## Supporting information

S1 Table Correlation analysis r values among diet ingredients, growth performance, diet (D) lipid classes, diet fatty acid composition, muscle (M) lipid classes, and muscle fatty acid composition (data with*, **, and *** represent P≤0.05, P ≤0.01, and P≤0.001, respectively). (DOCX)

S2 Table Correlation analysis r values among diet ingredients, diet lipid classes, diet (D) fatty acid composition, muscle (M) lipid classes, and muscle fatty acid composition (data with*, **, and *** represent P≤0.05, P ≤0.01, and P≤0.001, respectively). (DOCX)

S1 Fig Regression analysis between vegetable oil percentage in the diet and hepatosomatic index. (TIF)

S2 Fig Regression analyses between amount of 20:5ω3 (a) and 22:6ω3 (b) in the diet and muscle tissue.
(TIF)
