# Supplementary material for: Minimizing marine ingredients in diets of farmed Atlantic salmon (Salmo salar): Effects on growth performance and muscle lipid and fatty acid composition
Source: PLoS One. 2018 Sep 21;13(9):e0198538. doi: 10.1371/journal.pone.0198538 (PMC6150467; doi:10.1371/journal.pone.0198538)
Supplement: S1 Table — (DOCX) [file pone.0198538.s001.docx]

S1 Table. Correlation analysis r values among diet ingredients, growth performance, diet (D) lipid classes, diet fatty acid composition, muscle (M) lipid classes, and muscle fatty acid composition (data with*, **, and *** represent P≤0.05, P ≤0.01, and P≤0.001, respectively)

| Fish performance and diet and fish composition | Final weight  (g) | Weight gain  (g) | HSI^1^  (%) | NQC^3^  (g) | Final length  (cm) | SGR^4^  (% day^-1^) | CF^5^  (g cm^-1^) |
| --- | --- | --- | --- | --- | --- | --- | --- |
| VSI (%) |  | 0.780^*^ |  |  |  |  |  |
| NQC (g) | 0.807^*^ | 0.829^*^ |  |  |  |  |  |
| Fish meal (%) |  |  | 0.881^**^ |  |  |  |  |
| Animal by-products (%) |  |  |  |  |  |  | -0.770^*^ |
| Vegetable protein (%) |  |  | -0.805^*^ |  |  |  |  |
| Fish oil (%) | 0.760^*^ | 0.759^*^ | 0.767^*^ |  |  |  |  |
| Vegetable oil (%) |  |  | -0.942^**^ |  |  |  |  |
| Digestible protein (g Kg^-1^) |  |  | 0.766^*^ |  |  |  |  |
| EPA+DHA (%) |  |  | 0.831^*^ |  |  |  |  |
| Total Lipid (M) | 0.891^**^ | 0.912^**^ |  | 0.768^*^ |  | 0.945^***^ |  |
| Triacylglycerol (M) | 0.914^**^ | 0.937^**^ |  | 0.900^**^ |  | 0.948^***^ |  |
| Phospholipid (M) |  |  | -0.819^*^ |  |  |  |  |
| Ʃ PUFA^6^ (M) | 0.880^**^ | 0.906^**^ |  | 0.756^*^ |  | 0.912^**^ |  |
| P/S^7^ (M) |  |  | -0.851^*^ |  |  |  |  |
| Ʃ ω3 (M) | 0.838^*^ | 0.867^*^ |  |  |  | 0.855^*^ |  |
| DHA/EPA (M) |  |  | -0.835^*^ |  |  |  |  |
| Triacylglycerol (D) |  |  | -0.836^*^ |  |  |  |  |
| 18:1ω9 (D) |  |  | -0.930^**^ |  |  |  |  |
| 18:3ω3 (D) |  |  | -0.938^**^ |  |  |  |  |
| 20:4ω6 (D) |  |  | 0.772^*^ |  |  |  |  |
| 20:5ω3 (D) |  |  | 0.799^*^ |  |  |  |  |
| 22:6ω3 (D) |  |  | 0.804^*^ |  |  |  |  |
| Ʃ MUFA (D) |  |  | -0.843^*^ |  |  |  |  |
| Ʃ ω3 (D) | 0.847^*^ | 0.853^*^ |  | 0.840^*^ |  | 0.822^*^ |  |

^1^ Hepatosomatic index

^2^ Viscerosomatic index

^3^ Norwegian quality cut

^4^ Specific growth rate

^5^ Condition factor

^6^Polyunsaturated fatty acids

^7^ Polyunsaturated/saturated fatty acids

The unit for all lipids and fatty acids compositions is mg g^-1^ ww
