# Supplementary material for: Minimizing marine ingredients in diets of farmed Atlantic salmon (Salmo salar): Effects on growth performance and muscle lipid and fatty acid composition
Source: PLoS One. 2018 Sep 21;13(9):e0198538. doi: 10.1371/journal.pone.0198538 (PMC6150467; doi:10.1371/journal.pone.0198538)
Supplement: S2 Table — (DOCX) [file pone.0198538.s002.docx]

S2 Table. Correlation analysis r values among diet ingredients, diet lipid classes, diet (D) fatty acid composition, muscle (M) lipid classes, and muscle fatty acid composition (data with*, **, and *** represent P≤0.05, P ≤0.01, and P≤0.001, respectively)

| Fish performance and diet and fish composition | 22:6ω3 (M) | Ʃ SFA (M) | Ʃ MUFA (M) | P/S (M) | DHA/EPA (M) |
| --- | --- | --- | --- | --- | --- |
| 22:6ω3 (M) |  |  |  |  |  |
| Ʃ SFA^1^ (M) | 0.977^***^ |  |  |  |  |
| Ʃ MUFA^2^ (M) | 0.860^*^ | 0.936^**^ |  |  |  |
| Ʃ PUFA^4^ (M) | 0.941^**^ | 0.982^***^ | 0.981^***^ |  |  |
| P/S ^4^ (M) |  |  |  |  |  |
| Ʃ ω3 (M) | 0.994^***^ | 0.994^***^ | 0.901^**^ |  |  |
| DHA/EPA (M) |  |  |  | 0.925^**^ |  |
| 20:4ω6 (D) |  |  |  |  | -0.864^*^ |
| 20:5ω3 (D) | 0.775^*^ | 0.754^*^ |  | -0.781^*^ | -0.951^***^ |
| 22:6ω3 (D) | 0.775^*^ | 0.756^*^ |  | -0.797^*^ | -0.958^***^ |
| Ʃ PUFA (D) |  |  |  |  |  |
| P/S (D) | 0.818^*^ |  |  |  |  |
| Ʃ ω3 (D) |  | 0.878^**^ | 0.806^*^ |  | -0.817^*^ |

^1^ Saturated fatty acids

^2^ Monounsaturated fatty acids

^3^ Polyunsaturated fatty acids

^4^ Polyunsaturated/saturated fatty acids

The unit for all lipids and fatty acids compositions is mg g^-1^ ww
