# Supplementary figures and images for: Minimizing marine ingredients in diets of farmed Atlantic salmon (Salmo salar): Effects on growth performance and muscle lipid and fatty acid composition
Source: PLoS One. 2018 Sep 21;13(9):e0198538. doi: 10.1371/journal.pone.0198538 (PMC6150467; doi:10.1371/journal.pone.0198538)

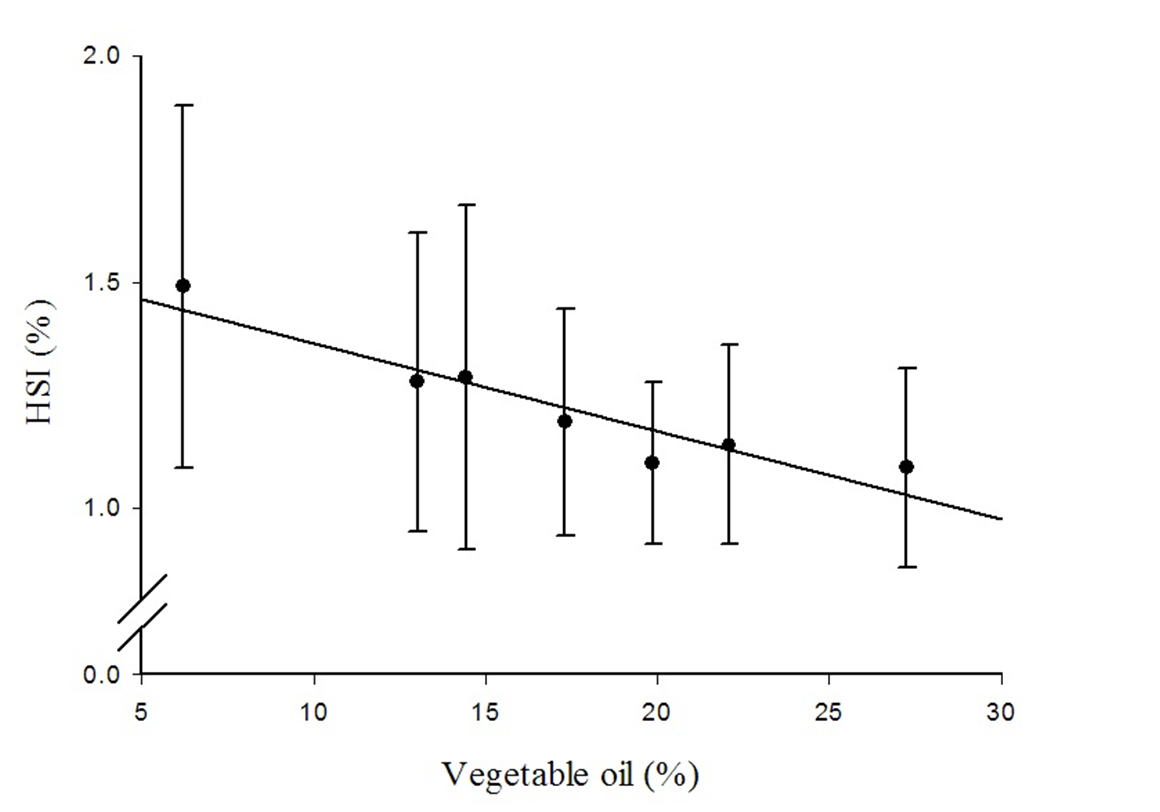

Supplement: S1 Fig — (TIF) [file pone.0198538.s003.tif]

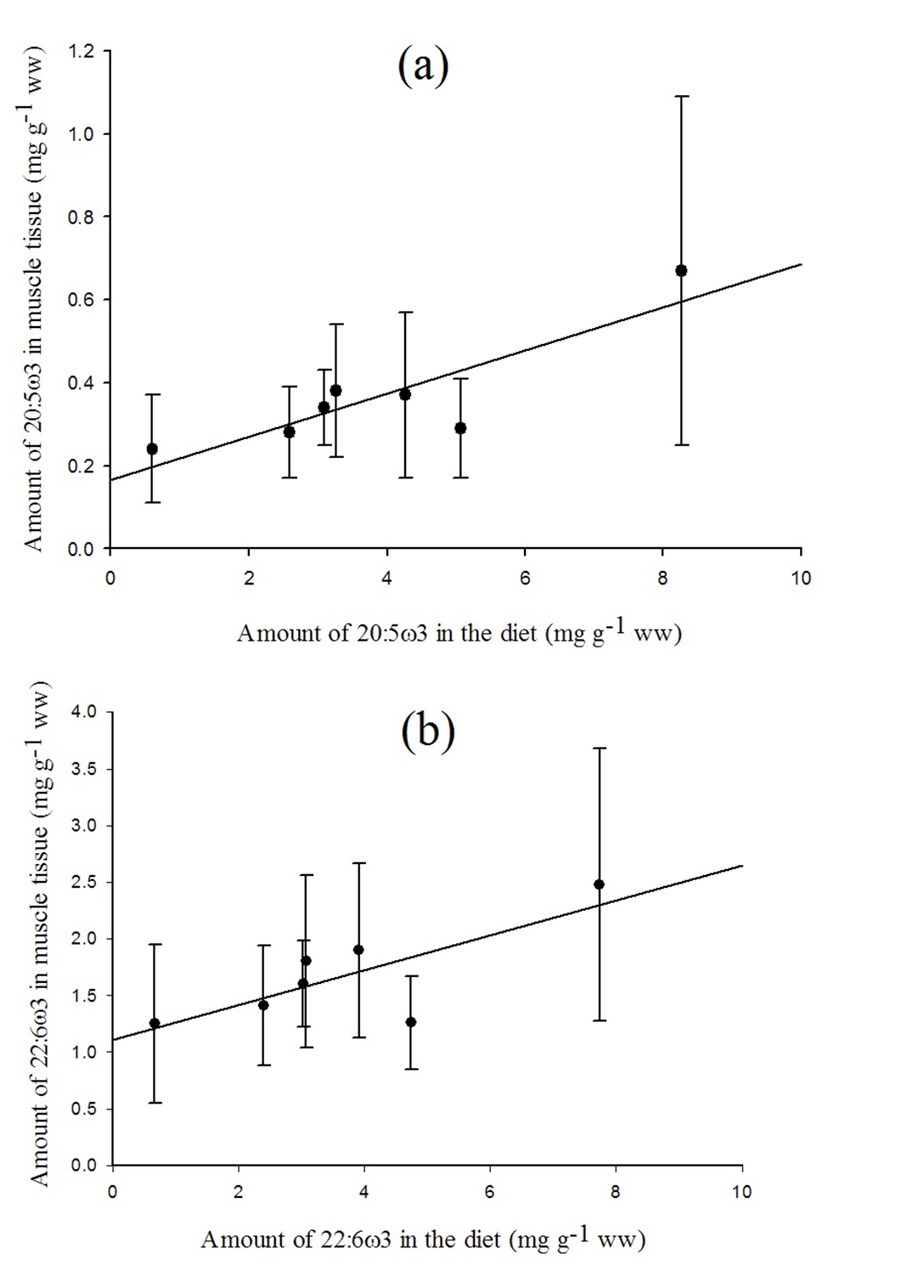

Supplement: S2 Fig — Regression analyses between amount of 20:5ω3 (a) and 22:6ω3 (b) in the diet and muscle tissue. (TIF) [file pone.0198538.s004.tif]
